# Understanding the endocannabinoid system as a modulator of the trigeminal pain response to concussion

**DOI:** 10.2217/cnc-2017-0010

**Published:** 2017-10-04

**Authors:** Melanie B Elliott, Sara J Ward, Mary E Abood, Ronald F Tuma, Jack I Jallo

**Affiliations:** 1 Department of Neurosurgery, Vickie & Jack Farber Institute for Neuroscience Thomas Jefferson University, PA 19107, USA; 2 Department of Pharmacology, Lewis Katz School of Medicine, Temple University, PA 19140, USA; 3 Department of Anatomy & Cell Biology, Lewis Katz School of Medicine, Temple University, PA 19140, USA; 4 Department of Physiology Lewis Katz School of Medicine, Temple University, PA 19140, USA

**Keywords:** cannabinoid, concussion trigeminal, endocannabinoid, post-traumatic headache, migraine

## Abstract

Post-traumatic headache is the most common symptom of postconcussion syndrome and becomes a chronic neurological disorder in a substantial proportion of patients. This review provides a brief overview of the epidemiology of postconcussion headache, research models used to study this disorder, as well as the proposed mechanisms. An objective of this review is to enhance the understanding of how the endogenous cannabinoid system is essential for maintaining the balance of the CNS and regulating inflammation after injury, and in turn making the endocannabinoid system a potential modulator of the trigeminal response to concussion. The review describes the role of endocannabinoid modulation of pain and the potential for use of phytocannabinoids to treat pain, migraine and concussion.

## Epidemiology of post-traumatic headache disorder

Post-traumatic headache disorders commonly share features with migraine or tension-type headache disorders and become chronic in a substantial portion of patients contributing to a poor quality of life and disability [1–5]. In the general population, the rates for persistent post-traumatic headache lasting a year or more are also exceedingly high, indicating a major health problem [6]. A significant subset (20%) of patients in the military with concussion have reported chronic daily headache [7]. According to the international classification of headache disorders-3 BETA, a diagnosis of persistent headache attributed to traumatic injury to the head is given when a headache persists for more than 3 months [8]. Adding to its burden, persistent headache after concussion or mild traumatic brain injury may contribute to poor sleep and psychosocial disorders, as well as complicate recovery from cognitive dysfunction after head injury [6].

This review provides a brief overview of the research models used to study postconcussion headache disorder, as well as the proposed mechanisms. Subsequently, the review covers the fundamentals of the endocannabinoid system (eCB) considering its relevance to migraine and traumatic brain injury research.

## Modeling post-traumatic headache

Migraine is the predominant chronic headache phenotype prevalent in 49–90% of military service members and nonmilitary patients with concussion or traumatic brain injury [1,6,9]. Diagnostic criteria for migraine includes sensitivity to light and sound (photophobia and phonophobia), nausea/vomiting, a unilateral headache, pulsate quality, moderate to severe intensity and headache aggravated by routine physical activity [5]. Mechanical allodynia, a cutaneous hypersensitivity to a mechanical stimulus, is a pain response common in patients with migraine, and has been reported in patients with post-traumatic headache [10–12]. Trigeminal or facial allodynia and photophobia behaviors have been used in animal models of head trauma and migraine to simulate clinical symptomology for studies of the mechanisms of post-traumatic headache [12–16]. Models of post-traumatic headache established by our laboratory show altered nociceptive signaling in the trigeminal pain pathway (increases in calcitonin gene-related peptide [CGRP] and nitric oxide synthase) in association with headache behaviors (facial allodynia and light sensitivity) [13–14,16]. In a more recent study by our lab, a model of postconcussion headache was used to assess parameters that have a significant impact on patient outcomes: the effects of head injury frequency and recovery time between injuries on the trigeminal pain system [17]. Other laboratories have used models of single mild head injury to study mechanisms of altered trigeminal pain signaling in the acute injury phase further expanding on the research and knowledge of this underserved problem [18–22]. There are differences in the methodologies among the laboratories to study post-traumatic headache using either a closed-head injury or fluid-percussion injury models to induce mild traumatic brain injury [17,18,21,22]. The current concussion models were derived from models dating back to the late eighties and modified to induce milder injuries which more accurately simulate the clinical features of the mild traumatic brain injury since mild traumatic brain injury accounts for the majority of traumatic brain injury [23–26].

## Pathophysiology of post-traumatic headache

A migraine attack involves the activation of the trigeminal pain pathway, in which the sensitization of either the peripheral pain neurons, central pain neurons or both has occurred (peripheral or central sensitization) [27]. Post-traumatic headache is believed to be due to inflammation-induced sensitization of trigeminal pain neurons located peripherally in the trigeminal ganglia and/or centrally in the caudal brain stem trigeminal nucleus caudalis, thalamic relay ventral posterior medialis nucleus or sensory cortex [13–14,18–19]. Concussions, as with other types of traumatic brain injuries, are heterogeneous injuries in that the injury location, impact force, history of concussion, age, gender and genetic factors will ultimately contribute to the effects on the central pain nuclei in the trigeminal system. Extracranial structures such as those in the meninges, periosteum, cranium and neck are innervated by the trigeminal ganglia or have ganglia that converge in the trigeminal pathway; these structures are a potential source of inflammation after concussion in addition to the intracranial structures of the trigeminal pathway. Immediately after concussion, inflammation of the meninges, particularly the dura, triggers activation and sensitization of the meningeal nociceptors [28–31]. Periosteal inflammation has also been shown to contribute to activation of the trigeminal ganglia neurons after head injury [19,32].

Initially, the release of inflammatory mediators such as cytokines, prostaglandins, nitric oxide (NO), bradykinins and histamine sensitize nociceptors and central trigeminal pain neurons leading to the development of chronic headache after head trauma [13,14,18,19,27,33]. Localized release of inflammatory mediators or sensitizers lower the threshold for the activation of meningeal nociceptors, and consequently increases the release of neuropeptides in response to an even smaller degree of vessel dilation or other stimulus [33,34]. CGRP and NO/nitric oxide synthase (NOS) are key pain signaling molecules that play an important role in the development of post-traumatic headache pathophysiology, as has been found for migraine [13,35–37]. Increases in CGRP and inducible NOS in the trigeminal pain pathway correlated with trigeminal allodynia [13,14,38] after cortical contusion and in a model of postconcussion headache [17]. Findings indicate either meningeal and/or periosteal inflammation sensitized the trigeminal nociceptors after head injury.

Several potential triggers have been noted to cause the release of CGRP that may play a role in post-traumatic headache; anandamide, an endogenous cannabinoid (CB) and ligand of the transient receptor potential channel, TRPV1 receptor, is released on demand after injury and triggers the release of CGRP [39]. TRPV1 receptors may be activated after head injury by way of cortical spreading depression (CSD), the spreading depolarization of cortical neurons, which is an accepted pathophysiological mechanism underlying the migraine aura [40]. Furthermore, serotonergic (5-HT) neurotransmission is another well-known mechanism in migraine pathophysiology in which triptans and some serotonin 5-HT uptake inhibitors are effective migraine treatments for many patients. Although it is typically thought that migraine involves a 5-HT deficiency, evidence also supports the hypothesis of 5-HT facilitated CSD in which increased cortical excitability may enhance trigeminal nociception [41].

Microglia have been implicated in the pathophysiological mechanisms of both migraine and chronic pain conditions [42,43]. Inhibition of microglia by minocycline reduces hyperalgesia induced by orofacial inflammation [44,45]. Our laboratory and others have shown that the transition of microglial cells to a pro-inflammatory M1 phenotype, is a predominant source of prolonged inflammation persisting after traumatic brain injury near the site of injury, the primary somatosensory cortex [13,46]. M1 microglia cells release pro-inflammatory and pro-nociceptive mediators (e.g., cytokines, chemokines, NO, prostaglandins and glutamate), and also upregulate CB receptors in response to injury [47,48] that may contribute to neuronal sensitization and chronic pain conditions including persistent post-traumatic headache [43,49–51]. Interestingly, the proliferation of microglia was associated with trigeminal sensitivity following repetitive mild head injury [17]. Findings of microglial proliferation and phenotype changes in the central trigeminal pain regions in areas remote from mechanical injury, such as the caudal brain stem, that lack evidence of axonal injury or signs of cell death may suggest that they may be serving a different function in pain processing than the classical M1 microglial function [13,16,17].

Astrocytes may also play a role in mediating post-traumatic headache. Previously, we showed the presence of delayed astrocytosis in the thalamus, an area involved in relaying pain, after a cortical injury in mice [38]. Astrocytosis in a model of post-concussion headache may indicate hyperexcitatory mechanisms that are involved in initiating acute post-traumatic headache [17]. Chronic pain models provide support for the role of glutamate regulation in the dorsal horn of the ascending pain pathway, which is predominantly handled by astrocytes [52]. An investigative team at the forefront of traumatic brain injury (TBI) research found treatment with a nonpsychotropic CB-based NMDA receptor antagonist improved outcomes following closed-head injury [26]. This research was timely, and although NMDA antagonist ultimately failed to show efficacy for neuroprotection, the sensory effects of head trauma including neuropathic pain and headache were never tested. In addition, blood–brain barrier permeability changes, which can be included along with astrocytes as part of the neurovascular unit, have also been implicated in models of migraine and may also contribute to post-concussion headache pathophysiology [53].

Headache following concussion that persists even after inflammation has resolved indicates central sensitization or other mechanisms may contribute to the pain [14,28]. Evidence from models of TBI show progressive neurodegenerative processes involving pain pathways, or synaptic plasticity that may account for the trigeminal-related changes in sensory behavior after mild TBI [46,54,55]. Sensitization of thalamic neurons has also been reported to play a role in the spread of allodynia in migraine patients and a rodent model of migraine [12]. Post-traumatic migraine models which study the driving mechanisms for the persistence of headache behaviors remain a highly needed area of research for this disorder.

## The endocannabinoid system

The eCB is essential to CNS homeostasis and plays a significant role in the regulation of inflammation and pain; however, the response of the endogenous cannabinoid system in post-traumatic headache is unknown, and the mechanistic explanations by which eCBs and synthetic CBs may alter trigeminal pain neurotransmission remain underdeveloped. Therefore, the fundamentals of the eCB system are covered subsequently, along with preclinical research for eCBs in migraine and traumatic brain injury research.

The eCB is comprised endogenously produced CBs, their receptors, and the proteins contributing to their synthesis and degradation [56,57]. The two CB receptors that have been most extensively studied are the CB1 receptor and the CB2 receptor. Recently a third receptor, GPR55, has received considerable attention, especially regarding its role in regulating pain responses. The eCB ligands are derived from the enzymatic degradation of precursors in the cell membrane and are released immediately after their synthesis, as opposed to being stored in secretory vesicles, as is the case for the so-called classic neurotransmitters. The eCB ligands first to be identified and most comprehensively studied are arachidonic acid (AA) metabolites N-arachidonylethanolamide, named ‘anandamide’ (AEA) from the Sanskrit word for ‘bliss’ [58], and a second metabolite 2-arachidonoylglycerol (2-AG) [59,60]. While AEA is primarily active at the CB1 receptor, 2-AG is active at both the CB1 and CB2 receptor. Biosynthesis of AEA is thought to be the result of enzymatic hydrolysis catalyzed by a phospholipase D of a membrane lipid precursor, *N*-arachidonoyl phosphatidylethanolamide. 2-AG is generated from diacylglycerol by diacylglycerol lipase. They are also rapidly inactivated by enzymatic degradation. The enzymes primarily responsible for their degradation are fatty acid amide hydrolase (FAAH) and monoacylglycerol lipase (MAGL) respectively. Cyclooxygenase-2 (COX-2) can also contribute to the degradation of both ligands. Arachidonate is a product of metabolism of both endogenous ligands, creating potential for significant interactions between the endocannabinoid and eicosanoid systems. Included among other endocannabinoid ligands are 2-arachidonyl glycerol ether (noladin ether), N-arachidonoyl-dopamine, oleoethanolamide, palmitoylethanolamide and virodhamine.

A common link between migraine and postconcussion headache may be in how endocannabinoids modulate the production of nitric oxide,   CGRP and 5-HT [61]. A reduction in AEA levels was found in the cerebral spinal fluid of patients with chronic migraine and correlated with increased C and products of nitric oxide [62]. This lead to an endocannabinoid deficiency as a proposed mechanism contributing to migraine disorders [63], and may also apply to post-traumatic headache. In a preclinical model of migraine, both degradative enzymes (FAAH and MAGL) were increased in the mesencephalon, while only FAAH was increased in the medulla. Treatment with AEA reduced nitroglycerin-induced hyperalgesia and neuronal activation in a model of migraine [64]. AEA combined with TRPV1 receptor inhibition reduced A and C fiber firing in the trigeminocervical complex induced by electrical stimulation of the dura [65]. Migraine research has provided initial evidence of altered eCBs and eCB degradation as a potential target for treatment of this disease.

In preclinical models of concussion, AEA increases in the injured cortex without changes in 2-AG in two different models of TBI, a neonatal rat model of weight drop closed-head injury and mouse model of moderate focal-cortical injury [66,67]. On the other hand, increases in 2-AG were reported for a mouse model of weight drop closed-head injury [68]. Rodent species or analysis methods for eCB differences may account for the differences reported for 2-AG after injury at the 24 h time points. The TBI models showing changes in eCBs were primarily induced by moderate injury as compared with the mild concussion injury models that have been developed more recently. For example, the models of weight drop closed-head injury may result in focal ischemic injury, immediate neuronal cell death and brain swelling, which would be present on neuroimaging [68]. An early study using the weight drop closed-head injury model reported a high early mortality rate due to apnea or secondary brain injury [69]. Concussion models would be expected to lack notable findings on neuroimaging, and although diffuse axonal injury and mild swelling may occur, focal damage is not typical of a mild concussion unless more severe or complicated. To date, there are no reports to our knowledge showing direct changes in the endocannabinoid system for the trigeminal pathway after mild concussion. One study by Zhang *et al*. reported MAGL inhibition was neuroprotective after repetitive mild closed-head injury [70]. Whether either AEA or 2-AG are increased acutely following concussion remains to be shown, however, if so, eCBs may serve to protect from inflammation, axonal injury/degenerative processes or secondary effects of altered glutamatergic excitatory transmission. Whether concussion affects the triggered release of eCBs later after injury demonstrated as a deficiency as proposed for migraine remains to be seen; further, when whether added triggers such as occurs during times of stress are areas needing to be addressed particularly when considering common comorbidities such as post-traumatic stress.

CB1, CB2 and GPR55 all appear to play an important role in the modulation of pain. The importance of the eCB for homeostasis is reflected by the fact that the CB1 receptor is the most abundant GPCR in the CNS. The CB1 CB receptor was discovered [71] and subsequently cloned [72] on the basis of its responsiveness to (-)-Δ9-tetrahydrocannabinol (Δ^9^-THC). Δ^9^-THCΔ9-THC). Δ9-THC is the primary psychoactive constituent in *Cannabis* (a.k.a. marijuana), hence the name ‘cannabinoid’ receptor. While there are constituently high levels of CB1 receptor expression in the CNS, the expression of the CB2 receptor is limited, but rapidly upregulates following trauma or inflammatory responses [73–77]. In the CNS, the CB1 receptor is found primarily on the presynaptic membrane of neurons, where it functions as the receptor for retrograde transmission of endogenous CBs released at the synapse, serving as a negative feedback modulator of synaptic transmission. It is primarily coupled to G_i/o_ proteins, causing inhibition of adenylyl cyclase, and influences numerous transcription factors and potassium channels. Retrograde signaling through the CB1 receptor modulates activity of both glutamatergic and GABA ergic neurons. There are also reports of modulation of other neurotransmitters. In this way, endoCB signaling can play an important role in both short- and long-term plasticity at both excitatory and inhibitory synapses. By modulating synaptic strength in these ways, the endoCB system can regulate a wide range of neuronal function including cognition, motor control, feeding behaviors and pain.

A second CB receptor (CB2), isolated by a PCR-based strategy designed to isolate GPCRs in differentiated myeloid cells, is often incorrectly stated to not be found within the brain or spinal cord [78]. Although their distribution on neurons is limited to a few selected sites, CB2 receptors are found on microglia, astrocytes and endothelial cells within the CNS. CB2 receptor agonist administration inhibits pain behaviors in models of peripheral inflammatory and neuropathic pain [79–81]. Our laboratory found the CB2 receptor agonist, JWH-133, abolished trigeminal allodynia and peptidergic signaling in the trigeminal pathway after cortical contusion in mice [82]. In a model of migraine, CB2 receptor stimulation resulted in short-term analgesia [83]. CB2 receptor activation has a well-documented role on immunomodulation and anti-inflammatory pathways [84,85]. In a series of studies, our laboratory showed that CB2 receptor stimulation resulted in robust effects on microglial activity, inducible NOS, TNF-α and intracellular adhesion molecule expression after cortical injury [29–30,86–87]. This evidence suggests that the CB2 receptor plays a role in inflammatory pain responses and that endogenous or synthetic CBs targeting this receptor would be expected to provide therapeutic benefit.

The GPR55 is found throughout the brain on various cells including neurons and microglia, and was initially classified as a CB receptor due to its activation by CB1/CB2 receptor ligands; although, non-CB ligands such as L-α-lysophosphatidylinositol (LPI), a lysophospholipid, also activate this receptor [88]. The broad CNS distribution of GPR55 suggests its involvement in central physiology and pathology [89]; *in situ* hybridization studies in rats indicated expression in hippocampus, thalamus and regions of the midbrain [90]. LPI-induced stimulation of sensory afferents correlated with dose-dependent development of mechanical hypersensitivity, allodynia and hyperalgesia, which were partially mediated by GPR55 [91]. Studies from our laboratory found that GPR55 activation at central levels is pronociceptive [92], suggesting that interfering with GPR55 signaling in the periaqueductal (PAG) may promote analgesia. Upon intra-PAG microinjection, LPI reduces (by half) the nociceptive threshold in the hot plate test in the rat [92]. These effects are dependent on GPR55 activation, since they are abolished by pretreatment with ML-193, a selective GPR55 antagonist [92]. GPR55 is another candidate CB receptor with the potential to be involved in modulating the trigeminal response to concussion, however, studies in this area to date are limited.

The three CB receptors, CB1, CB2 and GPR55 differ from one another in their potential for exerting psychotropic effects and immunomodulatory actions; however, it should be emphasized that all three receptors contribute to nociceptive signaling and pain [57,75,79–81,92–97]. Therefore, all three receptors may play an important role in modulating post-traumatic headache, and in turn make them therapeutic targets of interest. Whether one CB receptor bears greater weight in tipping the balance of chronic pain for certain injuries or diseases may be identified using selective agonists/antagonists. On the other hand, studies of mixed CB ligands such as those found in medicinal marijuana plant extracts or other CB compounds without known CB receptor effects (e.g., cannabidiol [CBD]) may provide insights into how the endogenous system works cohesively to balance the effects of injury.

Interest continues in the inhibition of the enzymatic degradation of eCBs. Some proof of concept studies in animal models of chronic and inflammatory pain show efficacy for selective inhibitors of FAAH-1 and MAGL, responsible for most of AEA and 2-AG enzymatic hydrolysis, respectively [98,99]. There is evidence for the effectiveness of FAAH inhibitors when used acutely in models of migraine resulting from injection of a nitric oxide donor to activate the trigeminal pain system [100,101] However, controversy exists regarding adverse effects of enzyme inhibition since compensatory inactivation of the ligands by other enzymatic degradations are proposed to occur in studies of FAAH and MAGL inhibitors for pain [102]. MAGL participates in the generation of AA as the precursor for eicosanoids (such as PGE2 and F2a), and although some products can be proinflammatory and pronociceptive, others, such as prostacyclin I2, play important physiological functions where inhibition may produce adverse events [103]. Moreover, chronic administration of MAGL inhibitors has been reported to desensitize CB1 receptors, and result in tolerance [104].

## Phytocannabinoids

Along with the heightened awareness among Americans of the legalization of medical marijuana and use across the USA to treat a variety of diseases and conditions including chronic and severe pain, there has come the rapid growth of the medical marijuana industry. To date, the medical and scientific communities face the challenge of keeping pace with the demands of addressing unanswered questions regarding the optimal formulations and treatment strategies for specific diseases such as post-traumatic migraine or primary migraine disorders. The range of phytocannabinoids, or those CBs naturally occurring in the whole cannabis plant, provide a vast therapeutic potential, especially once our understanding of which CBs are best for specific diseases becomes more developed. Although preclinical scientific evidence exists for certain diseases and conditions to warrant the use of medical marijuana or its various constituents, studies for migraine and post-traumatic migraine are as limited as the clinical research in this area.

The whole cannabis plant has over 150 different varieties of CBs reported to date [105] including the two most well-known CBs, Δ9-tetrayhydrocannabinol (THC) and CBD. There are a multitude of plant varieties of *Cannabis sativa* plant species being cultivated with adjustable ratios of THC and CBD yielding proposed therapeutic effects. In addition to CBs found in the Cannabis plant, roughly 140 terpenoids have been identified with proposed synergistic interactions with CBs, as well as independent therapeutic potential [105]. THC, the constituent responsible for the mind-altering and intoxicating effects of Cannabis Sativa was isolated in 1964 [106], and subsequently found to exert effects at the CB1 and CB2 receptors. Thereafter, the eCB system characterization began to develop [58]. More recent attention has been given to the multifactorial and complex interactions between THC and eCBs (see review by Lu and Mackie) [107]. Δ9-THC stimulates the release of endogenous opioids and AEA [108]. On the other hand, THC is reported to antagonize CB1 receptor signaling when stimulated by 2-AG as observed in several systems under low receptor density conditions [107]. The CB1 receptor antagonist, rimonabant, did not exert an effect on withdrawal syndrome in humans taking moderate doses of THC. In contrast, rimonabant elicited a robust withdrawal syndrome after long-term administration of high-dose THC in rodents [107]. These studies suggest the efficacy and interactions of cannabis or THC with the eCB may be dependent on usage, as well as final potency of the CBs. A recent retrospective study found medical marijuana to effectively reduce the frequency of headaches in patients, however, various side-effects were reported depending on the type of marijuana [109]. In regard to migraine pain, THC stimulates 5-HT synthesis [110], is a 5HT1A agonist, and 5HT3 antagonist implicating its role in the treatment of migraine and pain with several other potential mechanisms of action [111].

CBD is a nonpsychoactive phytocannabinoid which binds to sites other than CB1 or CB2 receptors. CBD is the most abundant *Cannabis*-derived non-CB1/CB2 receptor ligand [112]. CBD has been demonstrated to lack the euphoric, cognitive impairing [113] and appetite stimulating [114] effects of THC in rodents. CBD as a monotherapy is currently in clinical trials for a range of indications from seizure disorders to substance abuse, graft rejection and generalized anxiety disorder. There are no direct clinical or animal studies to date that we are aware of addressing the effects of CBD on concussion, but several animal studies demonstrate that CBD decreases brain injury following stroke [115–118]. There is one published case study for trigeminal neuralgia describing a patient's excellent response to Sativex [119]. Sativex, a buccal spray therapeutic with a well-documented safety profile in humans, is approved in the EU and Canada for chronic pain associated with multiple sclerosis and cancer and contains equal doses of CBD and THC, as well as a range of terpenes and flavonoids contained in *Cannabis*. The work of our group and others has demonstrated in rodent models that CBD attenuates inflammatory and neuropathic pain [113,120–123]. There is supporting evidence for several mechanisms underlying these neuroprotective effects, including actions on 5-HT1A receptors, TRPV1 channels and α3 glycine receptors. CBD alone has not been directly tested in humans for the treatment of pain, but Sativex has produced significant pain-relieving effects in several clinical studies [124,125]. There are striking similarities and differences between CBD and direct CB receptor agonists. Like some other CBs, CBD suppresses the release of cytokines and chemokines, decreases production of reactive oxygen species, and attenuates immune cell proliferation, activation, maturation, migration and antigen presentation [126,127]. Dysregulation of these factors is thought to contribute to both acute dysfunction and to long term consequences of head injury including chronic pain and headache. Specifically, recent studies demonstrate that CBD attenuates microglial activation, immune cell migration and reactive oxygen species following a range of pro-inflammatory insults [127–129]. Importantly, the attenuation of lymphocyte activation by CBD was recently shown to be specific to B, T and Th lymphocytes, while CBD administration actually increased total numbers of natural killer cells [130], demonstrating that CBD is not panimmunosuppressive. CBD has also been shown to attenuate endothelial inflammation and blood–brain barrier disruption [131]. CBD reduced iNos expression in a mouse model of tauopathy with implications for concussions and chronic traumatic encephalopathy [132]. In fact, all aforementioned mechanisms of action for CBD have been proposed mechanisms for post-traumatic headache as reviewed, concussion or migraine pathophysiology. Taken together, this wide range of immunomodulatory and neurobehavioral effects points to CBD as an exciting potential pharmacotherapy in the treatment of concussion and other TBIs.

## Conclusion

Headache after concussion persists in a substantial portion of patients. Early inflammatory sources activate trigeminal ganglia neurons to release excitatory neuropeptides and neurotransmitters. Centralized sources of inflammation after concussion may sensitize neurons at several regions along the trigeminal nuclei. Sensitized trigeminal neurons lead to a lowered threshold of activation, which more readily triggers a headache. The eCB system is believed to contribute to the homeostatic basis by which both the CNS and immune system respond to injury, while also being a main modulator of the pain system. Whether there is a deficiency in the eCB after concussion as proposed for migraine has yet to be evidenced. Exogenous modulators of the eCB, whether derived from the plant or synthetics, have been shown to alter pain in acute, chronic and neuropathic conditions, including migraine and are expected to play a large role in the headache and other pain conditions resulting from concussion.

## Future perspective

Currently there are no US-FDA approved treatments for concussion and therapies used for post-traumatic headache are implemented based on primary headache disorders such as migraine or tension type headache. Increased research into the study of the eCB system and its modulators, particularly for underrepresented disorders such as post-traumatic headache and migraine, will facilitate making medical advances toward the development of more efficacious therapeutics for these disorders. Progress towards understanding the mechanisms of post-traumatic headache, although slow, is heading in the right direction as more researchers study the problem along with support through incremental increases in funding opportunities via multiple sources (i.e., DoD, NIH, foundations). Over the next five to ten years, once the barriers to research that impact the progress in the study of the endocannabinoid system and particularly phytocannabinoids for the treatment of headache and related symptoms (i.e., vertigo, fatigue, sleep dysfunction, cognitive and attentional deficits, depression and anxiety) following concussion be addressed will progress be made;  challenges impacting research include limited federal funding allocated for this research area, tight federal regulations and the supply of phytocannabinoids.

**Executive summary**

**Epidemiology of post-traumatic headache disorder**

- Persistent post-traumatic headache lasting a year or more are also exceedingly high, indicating a major health problem.
- Headache induced by concussion predominantly share features with migraine, followed by tension-type headache.

**Modeling post-traumatic headache**

- Models of mild closed-head injury or mild fluid-percussion injury have been used to demonstrate changes in the trigeminal pain sytem to study post-traumatic or post-concussion headache.

**Pathophysiology of post-traumatic headache**

- Evidence from animals models to date indicate acute inflammation stemming from the meninges, periosteal mast cells following concussion injury sensitizes the meningeal nociceptors facilitating head pain and cutanous sensitivity.
- Additional evidence suggests altered signaling in central pain regions that are not well characterized in current concussionmodelsmay also contribute to pain facilitation, ongoing headache and abnormal sensitivity.
- Microglia and astrocytes provide different cellular mechanisms of post-concussion headache including inflammation and excitatory processes; the persistence of headache after concussion beyond the acute period when inflammation has resolved points toward other mechanisms for the maintenance of pain.

**The endocannabinoid system**

- The eCB is essential to central nervous system homeostasis as well as the regulation of the immune and pain systems.
- Post-concussion headache and migraine may be linked in how eCBs modulation the production of certain neurotransmitters and second messangers.
- Expanding on the current data  showing changes in the eCB system in models of concussion and TBI to include the pain pathway is warranted.

**Phytocannabinoids**

- Phytocannabinoids derived from the whole plant, each with distinct chemical properties, have a unique therapeutic potential that may ultimately be identified as disease specific.

## References

[1] Lucas S, Hoffman JM, Bell KR, Dikmen S (2013). A prospective study of prevalence and characterization of headache following mild traumatic brain injury. *Cephalalgia*.

[2] Hoffman JM, Lucas S, Dikmen S (2011). Natural history of headache after traumatic brain injury. *J. Neurotrauma*.

[3] Lucas S (2011). Headache management in concussion and mild traumatic brain injury. *PM R*.

[4] Seifert TD (2013). Sports concussion and associated post-traumatic headache. *Headache*.

[5] Headache Classification Committee of the International Headache S (2013). The International Classification of Headache Disorders (3rd Edition) (beta version). *Cephalalgia*.

[6] Theeler B, Lucas S, Riechers RG, Ruff RL (2013). Post-traumatic headaches in civilians and military personnel: a comparative, clinical review. *Headache*.

[7] Theeler BJ, Erickson JC (2012). Posttraumatic headache in military personnel and veterans of the iraq and afghanistan conflicts. *Curr. Treat. Options Neurol.*.

[8] Ichd-3 (the International Classification of Headache Disorders RE (2013). Headache Classification Committee of the International Headache, Society. *Cephalalgia*.

[9] Theeler BJ, Flynn FG, Erickson JC (2010). Headaches after concussion in US soldiers returning from Iraq or Afghanistan. *Headache*.

[10] Ofek H, Defrin R (2007). The characteristics of chronic central pain after traumatic brain injury. *Pain*.

[11] Burstein R, Yarnitsky D, Goor-Aryeh I, Ransil BJ, Bajwa ZH (2000). An association between migraine and cutaneous allodynia. *Ann. Neurol.*.

[12] Burstein R, Jakubowski M, Garcia-Nicas E (2010). Thalamic sensitization transforms localized pain into widespread allodynia. *Ann. Neurol.*.

[13] Elliott MB, Oshinsky ML, Amenta PS, Awe OO, Jallo JI (2012). Nociceptive neuropeptide increases and periorbital allodynia in a model of traumatic brain injury. *Headache*.

[14] Macolino CM, Daiutolo BV, Alberston BK, Elliott MB (2014). Mechanical alloydnia induced by traumatic brain injury is independent of restraint stress. *J. Neurosci. Methods*.

[15] Recober A, Kuburas A, Zhang Z, Wemmie JA, Anderson MG, Russo AF (2009). Role of calcitonin gene-related peptide in light-aversive behavior: implications for migraine. *J. Neurosci.*.

[16] Daiutolo BV, Tyburski A, Clark SW, Elliott MB (2016). Trigeminal pain molecules, allodynia, and photosensitivity are pharmacologically and genetically modulated in a model of traumatic brain injury. *J. Neurotrauma*.

[17] Tyburski A, Cheng L, Assari S, Darvish K, Elliott M (2017). Frequent mild head injury promotes trigeminal sensitivity concomitant with microglial proliferation, astrocytosis, and increased neuropeptide levels in the trigeminal pain system. *J. Headache Pain*.

[18] Benromano T, Defrin R, Ahn AH, Zhao J, Pick CG, Levy D (2015). Mild closed head injury promotes a selective trigeminal hypernociception: implications for the acute emergence of post-traumatic headache. *Eur. J. Pain*.

[19] Feliciano DP, Sahbaie P, Shi X, Klukinov M, Clark JD, Yeomans DC (2014). Nociceptive sensitization and BDNF up-regulation in a rat model of traumatic brain injury. *Neurosci. Lett.*.

[20] Mustafa G, Hou J, Tsuda S (2016). Trigeminal neuroplasticity underlies allodynia in a preclinical model of mild closed head traumatic brain injury (cTBI). *Neuropharmacology*.

[21] Levy D, Edut S, Baraz-Goldstein R (2016). Responses of dural mast cells in concussive and blast models of mild traumatic brain injury in mice: potential implications for post-traumatic headache. *Cephalalgia*.

[22] Bree D, Levy D (2016). Development of CGRP-dependent pain and headache related behaviours in a rat model of concussion: implications for mechanisms of post-traumatic headache. *Cephalalgia*.

[23] Schmidt RH, Grady MS (1993). Regional patterns of blood–brain barrier breakdown following central and lateral fluid-percussion injury in rodents. *J. Neurotrauma*.

[24] Lighthall JW (1988). Controlled cortical impact: a new experimental brain injury model. *J. Neurotrauma*.

[25] Shapira Y, Setton D, Artru AA, Shohami E (1993). Blood–brain barrier permeability, cerebral edema, and neurologic function after closed head injury in rats. *Anesth. Analg.*.

[26] Shohami E, Novikov M, Mechoulam R (1993). A nonpsychotropic cannabinoid, HU-211, has cerebroprotective effects after closed head injury in the rat. *J. Neurotrauma*.

[27] Bernstein C, Burstein R (2012). Sensitization of the trigeminovascular pathway: perspective and implications to migraine pathophysiology. *J. Clin. Neurol.*.

[28] Woolf CJ, Salter MW (2000). Neuronal plasticity: increasing the gain in pain. *Science*.

[29] Amenta PS, Jallo JI, Tuma RF, Elliott MB (2012). A cannabinoid type 2 receptor agonist attenuates blood–brain barrier damage and neurodegeneration in a murine model of traumatic brain injury. *J. Neurosci. Res.*.

[30] Amenta PS, Jallo JI, Tuma RF, Hooper DC, Elliott MB (2014). Cannabinoid receptor type-2 stimulation, blockade, and deletion alters the vascular inflammatory responses to traumatic brain injury. *J. Neuroinflammation*.

[31] Woolf CJ, Ma Q (2007). Nociceptors–noxious stimulus detectors. *Neuron*.

[32] Benromano T, Defrin R, Ahn AH, Zhao J, Pick CG, Levy D (2014). Mild closed head injury promotes a selective trigeminal hypernociception: implications for the acute emergence of post-traumatic headache. *Eur. J. Pain*.

[33] Levy D, Kainz V, Burstein R, Strassman AM (2012). Mast cell degranulation distinctly activates trigemino-cervical and lumbosacral pain pathways and elicits widespread tactile pain hypersensitivity. *Brain. Behav. Immun.*.

[34] Levy D, Burstein R (2011). The vascular theory of migraine: leave it or love it?. *Ann. Neurol.*.

[35] Goadsby PJ, Edvinsson L, Ekman R (1990). Vasoactive peptide release in the extracerebral circulation of humans during migraine headache. *Ann. Neurol.*.

[36] Recober A, Goadsby PJ (2010). Calcitonin gene-related peptide: a molecular link between obesity and migraine?. *Drug News Perspect.*.

[37] Edvinsson L, Mulder H, Goadsby PJ, Uddman R (1998). Calcitonin gene-related peptide and nitric oxide in the trigeminal ganglion: cerebral vasodilatation from trigeminal nerve stimulation involves mainly calcitonin gene-related peptide. *J. Auton. Nerv. Syst.*.

[38] Hazra A, Macolino C, Elliott MB, Chin J (2014). Delayed thalamic astrocytosis and disrupted sleep-wake patterns in a preclinical model of traumatic brain injury. *J. Neurosci. Res.*.

[39] Akerman S, Kaube H, Goadsby PJ (2003). Vanilloid type 1 receptors (VR1) on trigeminal sensory nerve fibres play a minor role in neurogenic dural vasodilatation, and are involved in capsaicin-induced dural dilation. *Br. J. Pharmacol.*.

[40] Meents JE, Neeb L, Reuter U (2010). TRPV1 in migraine pathophysiology. *Trends Mol. Med.*.

[41] Hamel E (2007). Serotonin and migraine: biology and clinical implications. *Cephalalgia*.

[42] Grinberg YY, Milton JG, Kraig RP (2011). Spreading depression sends microglia on Levy flights. *PLoS ONE*.

[43] Watkins LR, Milligan ED, Maier SF (2001). Glial activation: a driving force for pathological pain. *Trends Neurosci.*.

[44] Henry CJ, Huang Y, Wynne AM, Godbout JP (2009). Peripheral lipopolysaccharide (LPS) challenge promotes microglial hyperactivity in aged mice that is associated with exaggerated induction of both pro-inflammatory IL-1beta and anti-inflammatory IL-10 cytokines. *Brain. Behav. Immun.*.

[45] Shimizu K, Guo W, Wang H (2009). Differential involvement of trigeminal transition zone and laminated subnucleus caudalis in orofacial deep and cutaneous hyperalgesia: the effects of interleukin-10 and glial inhibitors. *Mol. Pain*.

[46] Onyszchuk G, Levine SM, Brooks WM, Berman NE (2009). Post-acute pathological changes in the thalamus and internal capsule in aged mice following controlled cortical impact injury: a magnetic resonance imaging, iron histochemical, and glial immunohistochemical study. *Neurosci. Lett.*.

[47] Romero-Sandoval A, Nutile-Mcmenemy N, Deleo JA (2008). Spinal microglial and perivascular cell cannabinoid receptor type 2 activation reduces behavioral hypersensitivity without tolerance after peripheral nerve injury. *Anesthesiology*.

[48] Wu J, Bie B, Yang H, Xu JJ, Brown DL, Naguib M (2013). Activation of the CB2 receptor system reverses amyloid-induced memory deficiency. *Neurobiol. Aging*.

[49] Deleo JA, Yezierski RP (2001). The role of neuroinflammation and neuroimmune activation in persistent pain. *Pain*.

[50] Scholz J, Woolf CJ (2007). The neuropathic pain triad: neurons, immune cells and glia. *Nat. Neurosci.*.

[51] Wieseler-Frank J, Maier SF, Watkins LR (2004). Glial activation and pathological pain. *Neurochem. Int.*.

[52] Tao YX, Gu J, Stephens RL (2005). Role of spinal cord glutamate transporter during normal sensory transmission and pathological pain states. *Mol. Pain*.

[53] Fried NT, Maxwell CR, Elliott MB, Oshinsky ML (2017). Region-specific disruption of the blood–brain barrier following repeated inflammatory dural stimulation in a rat model of chronic trigeminal allodynia. *Cephalalgia*.

[54] Hall ED, Bryant YD, Cho W, Sullivan PG (2008). Evolution of post-traumatic neurodegeneration after controlled cortical impact traumatic brain injury in mice and rats as assessed by the de Olmos silver and fluorojade staining methods. *J. Neurotrauma*.

[55] Hall KD, Lifshitz J (2010). Diffuse traumatic brain injury initially attenuates and later expands activation of the rat somatosensory whisker circuit concomitant with neuroplastic responses. *Brain Res.*.

[56] Di Marzo V, Melck D, Bisogno T, De Petrocellis L (1998). Endocannabinoids: endogenous cannabinoid receptor ligands with neuromodulatory action. *Trends Neurosci.*.

[57] Pacher P, Batkai S, Kunos G (2006). The endocannabinoid system as an emerging target of pharmacotherapy. *Pharmacol. Rev.*.

[58] Devane WA, Hanus L, Breuer A (1992). Isolation and structure of a brain constituent that binds to the cannabinoid receptor. *Science*.

[59] Mechoulam R, Ben-Shabat S, Hanus L (1995). Identification of an endogenous 2-monoglyceride, present in canine gut, that binds to cannabinoid receptors. *Biochem. Pharmacol.*.

[60] Sugiura T, Kondo S, Sukagawa A (1995). 2-Arachidonoylglycerol: a possible endogenous cannabinoid receptor ligand in brain. *Biochem. Biophys. Res. Commun.*.

[61] Pertwee RG (2001). Cannabinoid receptors and pain. *Progr. Neurobiol.*.

[62] Sarchielli P, Pini LA, Coppola F (2007). Endocannabinoids in chronic migraine: CSF findings suggest a system failure. *Neuropsychopharmacology*.

[63] Russo EB (2004). Clinical endocannabinoid deficiency (CECD): can this concept explain therapeutic benefits of cannabis in migraine, fibromyalgia, irritable bowel syndrome and other treatment-resistant conditions?. *Neuro Endocrinol. Lett.*.

[64] Greco R, Mangione AS, Sandrini G, Maccarrone M, Nappi G, Tassorelli C (2011). Effects of anandamide in migraine: data from an animal model. *J. Headache Pain*.

[65] Akerman S, Holland PR, Goadsby PJ (2007). Cannabinoid (CB1) receptor activation inhibits trigeminovascular neurons. *J. Pharmacol. Exp. Ther.*.

[66] Hansen HH, Schmid PC, Bittigau P (2001). Anandamide, but not 2-arachidonoylglycerol, accumulates during *in vivo* neurodegeneration. *J. Neurochem.*.

[67] Tchantchou F, Tucker LB, Fu AH (2014). The fatty acid amide hydrolase inhibitor PF-3845 promotes neuronal survival, attenuates inflammation and improves functional recovery in mice with traumatic brain injury. *Neuropharmacology*.

[68] Panikashvili D, Simeonidou C, Ben-Shabat S (2001). An endogenous cannabinoid (2-AG) is neuroprotective after brain injury. *Nature*.

[69] Chen Y, Constantini S, Trembovler V, Weinstock M, Shohami E (1996). An experimental model of closed head injury in mice: pathophysiology, histopathology, and cognitive deficits. *J. Neurotrauma*.

[70] Zhang J, Teng Z, Song Y, Hu M, Chen C (2015). Inhibition of monoacylglycerol lipase prevents chronic traumatic encephalopathy-like neuropathology in a mouse model of repetitive mild closed head injury. *J. Cereb. Blood Flow Metab.*.

[71] Devane WA, Dysarz FA, Johnson MR, Melvin LS, Howlett AC (1988). Determination and characterization of a cannabinoid receptor in rat brain. *Mol. Pharmacol.*.

[72] Matsuda LA, Lolait SJ, Brownstein MJ, Young AC, Bonner TI (1990). Structure of a cannabinoid receptor and functional expression of the cloned cDNA. *Nature*.

[73] Alonso-Ferrero ME, Paniagua MA, Mostany R (2006). Cannabinoid system in the budgerigar brain. *Brain Res.*.

[74] Rodriguez De, Fonseca F, Del Arco I, Bermudez-Silva FJ, Bilbao A, Cippitelli A, Navarro M (2005). The endocannabinoid system: physiology and pharmacology. *Alcohol Alcohol.*.

[75] Van Sickle MD, Duncan M, Kingsley PJ (2005). Identification and functional characterization of brainstem cannabinoid CB2 receptors. *Science*.

[76] Ashton JC (2011). The use of knockout mice to test the specificity of antibodies for cannabinoid receptors. *Hippocampus*.

[77] Atwood BK, Mackie K (2010). CB2: a cannabinoid receptor with an identity crisis. *Br. J. Pharmacol.*.

[78] Munro S, Thomas KL, Abu-Shaar M (1993). Molecular characterization of a peripheral receptor for cannabinoids. *Nature*.

[79] Yamamoto W, Mikami T, Iwamura H (2008). Involvement of central cannabinoid CB2 receptor in reducing mechanical allodynia in a mouse model of neuropathic pain. *Eur. J. Pharmacol.*.

[80] Beltramo M, Bernardini N, Bertorelli R (2006). CB2 receptor-mediated antihyperalgesia: possible direct involvement of neural mechanisms. *Eur. J. Neurosci.*.

[81] Ibrahim MM, Rude ML, Stagg NJ (2006). CB2 cannabinoid receptor mediation of antinociception. *Pain*.

[82] Macolino CB, Tyburski AL, Albertson BK, Hooper DC, Elliott MB (2013). The selective cannabinoid type-2 receptor agonist, JWH-133, abolishes mechanical alloydnia in a model of post-traumatic headache. *Cephalalgia*.

[83] Greco R, Mangione AS, Sandrini G, Nappi G, Tassorelli C (2014). Activation of CB2 receptors as a potential therapeutic target for migraine: evaluation in an animal model. *J. Headache Pain*.

[84] Cabral GA, Marciano-Cabral F (2005). Cannabinoid receptors in microglia of the central nervous system: immune functional relevance. *J. Leukoc. Biol.*.

[85] Carrier EJ, Patel S, Hillard CJ (2005). Endocannabinoids in neuroimmunology and stress. *Curr. Drug Targets CNS Neurol. Disord.*.

[86] Elliott MB, Tuma RF, Amenta PS, Barbe MF, Jallo JI (2011). Acute effects of a selective cannabinoid-2 receptor agonist on neuroinflammation in a model of traumatic brain injury. *J. Neurotrauma*.

[87] Elliott MB, Tuma RF, Amenta PS, Barbe MF, Jallo JI (2011). Acute effects of a selective cannabinoid-2 receptor agonist on neuroinflammation in a murine model of traumatic brain injury. *J. Neurotrauma*.

[88] Kotsikorou E, Sharir H, Shore DM (2013). Identification of the GPR55 antagonist binding site using a novel set of high-potency GPR55 selective ligands. *Biochemistry*.

[89] Henstridge CM (2012). Off-target cannabinoid effects mediated by GPR55. *Pharmacology*.

[90] Sawzdargo M, Nguyen T, Lee DK (1999). Identification and cloning of three novel human G protein-coupled receptor genes GPR52, PsiGPR53 and GPR55: GPR55 is extensively expressed in human brain. *Brain Res. Mol. Brain Res.*.

[91] Gangadharan V, Selvaraj D, Kurejova M (2013). A novel biological role for the phospholipid lysophosphatidylinositol in nociceptive sensitization via activation of diverse G-protein signalling pathways in sensory nerves *in vivo*. *Pain*.

[92] Deliu E, Sperow M, Console-Bram L (2015). The Lysophosphatidylinositol Receptor GPR55 Modulates Pain Perception in the Periaqueductal Gray. *Mol. Pharmacol.*.

[93] Wu CS, Chen H, Sun H (2013). GPR55, a G-protein coupled receptor for lysophosphatidylinositol, plays a role in motor coordination. *PLoS ONE*.

[94] Klein TW (2005). Cannabinoid-based drugs as anti-inflammatory therapeutics. *Nat. Rev. Immunol.*.

[95] Klein TW, Cabral GA (2006). Cannabinoid-induced immune suppression and modulation of antigen-presenting cells. *J. Neuroimmune Pharmacol.*.

[96] Mackie K (2006). Cannabinoid receptors as therapeutic targets. *Annu. Rev. Pharmacol. Toxicol.*.

[97] Pertwee RG, Ross RA (2002). Cannabinoid receptors and their ligands. *Prostaglandins Leukot. Essent. Fatty Acids*.

[98] Di Marzo V (2008). Targeting the endocannabinoid system: to enhance or reduce?. *Nat. Rev. Drug Discov.*.

[99] Feledziak M, Lambert DM, Marchand-Brynaert J, Muccioli GG (2012). Inhibitors of the endocannabinoid-degrading enzymes, or how to increase endocannabinoid's activity by preventing their hydrolysis. *Recent Pat. CNS Drug Discov.*.

[100] Greco R, Bandiera T, Mangione AS (2015). Effects of peripheral FAAH blockade on NTG-induced hyperalgesia–evaluation of URB937 in an animal model of migraine. *Cephalalgia*.

[101] Nozaki C, Markert A, Zimmer A (2015). Inhibition of FAAH reduces nitroglycerin-induced migraine-like pain and trigeminal neuronal hyperactivity in mice. *Eur. Neuropsychopharmacol.*.

[102] Maione S, Costa B, Di Marzo V (2013). Endocannabinoids: a unique opportunity to develop multitarget analgesics. *Pain*.

[103] Nomura DK, Morrison BE, Blankman JL (2011). Endocannabinoid hydrolysis generates brain prostaglandins that promote neuroinflammation. *Science*.

[104] Schlosburg JE, Blankman JL, Long JZ (2010). Chronic monoacylglycerol lipase blockade causes functional antagonism of the endocannabinoid system. *Nat. Neurosci.*.

[105] Giese MW, Lewis MA, Giese L, Smith KM (2015). Development and validation of a reliable and robust method for the analysis of cannabinoids and terpenes in cannabis. *J. AOAC Int.*.

[106] Mechoulam R, Gaoni Y (1965). A total synthesis of Dl-delta-1-tetrahydrocannabinol, the active constituent of hashish. *J. Am. Chem. Soc.*.

[107] Lu HC, Mackie K (2016). An introduction to the endogenous cannabinoid system. *Biol. Psychiatry*.

[108] Welch SP (2009). Interaction of the cannabinoid and opioid systems in the modulation of nociception. *Int. Rev. Psychiatry*.

[109] Rhyne DN, Anderson SL, Gedde M, Borgelt LM (2016). Effects of medical marijuana on migraine headache frequency in an adult population. *Pharmacotherapy*.

[110] Russo EB (2008). Clinical endocannabinoid deficiency (CECD): can this concept explain therapeutic benefits of cannabis in migraine, fibromyalgia, irritable bowel syndrome and other treatment-resistant conditions?. *Neuro Endocrinol. Lett.*.

[111] Baron EP (2015). Comprehensive review of medicinal marijuana, cannabinoids, and therapeutic implications in medicine and headache: what a long strange trip it's been. *Headache*.

[112] Showalter VM, Compton DR, Martin BR, Abood ME (1996). Evaluation of binding in a transfected cell line expressing a peripheral cannabinoid receptor (CB2): identification of cannabinoid receptor subtype selective ligands. *J. Pharmacol. Exp. Ther.*.

[113] Ward SJ, Mcallister SD, Kawamura R, Murase R, Neelakantan H, Walker EA (2014). Cannabidiol inhibits paclitaxel-induced neuropathic pain through 5-HT(1A) receptors without diminishing nervous system function or chemotherapy efficacy. *Br. J. Pharmacol.*.

[114] Riedel G, Fadda P, Mckillop-Smith S, Pertwee RG, Platt B, Robinson L (2009). Synthetic and plant-derived cannabinoid receptor antagonists show hypophagic properties in fasted and non-fasted mice. *Br. J. Pharmacol.*.

[115] Hayakawa K, Mishima K, Abe K (2004). Cannabidiol prevents infarction via the non-CB1 cannabinoid receptor mechanism. *Neuroreport*.

[116] Hayakawa K, Mishima K, Nozako M (2007). Delayed treatment with cannabidiol has a cerebroprotective action via a cannabinoid receptor-independent myeloperoxidase-inhibiting mechanism. *J. Neurochem.*.

[117] Mishima K, Hayakawa K, Abe K (2005). Cannabidiol prevents cerebral infarction via a serotonergic 5-hydroxytryptamine1A receptor-dependent mechanism. *Stroke*.

[118] Hind WH, England TJ, O'sullivan SE (2016). Cannabidiol protects an *in vitro* model of the blood–brain barrier from oxygen-glucose deprivation via PPARgamma and 5-HT1A receptors. *Br. J. Pharmacol.*.

[119] Gajofatto A (2016). Refractory trigeminal neuralgia responsive to nabiximols in a patient with multiple sclerosis. *Mult. Scler. Relat. Disord.*.

[120] Ward SJ, Ramirez MD, Neelakantan H, Walker EA (2011). Cannabidiol prevents the development of cold and mechanical allodynia in paclitaxel-treated female C57Bl6 mice. *Anesth. Analg.*.

[121] Costa B, Trovato AE, Comelli F, Giagnoni G, Colleoni M (2007). The non-psychoactive cannabis constituent cannabidiol is an orally effective therapeutic agent in rat chronic inflammatory and neuropathic pain. *Eur. J. Pharmacol.*.

[122] Comelli F, Giagnoni G, Bettoni I, Colleoni M, Costa B (2008). Antihyperalgesic effect of a Cannabis sativa extract in a rat model of neuropathic pain: mechanisms involved. *Phytother. Res.*.

[123] Xiong W, Cui T, Cheng K (2012). Cannabinoids suppress inflammatory and neuropathic pain by targeting alpha3 glycine receptors. *J. Exp. Med.*.

[124] Nurmikko TJ, Serpell MG, Hoggart B, Toomey PJ, Morlion BJ, Haines D (2007). Sativex successfully treats neuropathic pain characterised by allodynia: a randomised, double-blind, placebo-controlled clinical trial. *Pain*.

[125] Iskedjian M, Bereza B, Gordon A, Piwko C, Einarson TR (2007). Meta-analysis of cannabis based treatments for neuropathic and multiple sclerosis-related pain. *Curr. Med. Res. Opin.*.

[126] Lodzki M, Godin B, Rakou L, Mechoulam R, Gallily R, Touitou E (2003). Cannabidiol-transdermal delivery and anti-inflammatory effect in a murine model. *J. Control. Release*.

[127] Mechoulam R, Peters M, Murillo-Rodriguez E, Hanus LO (2007). Cannabidiol–recent advances. *Chem. Biodivers.*.

[128] Rimmerman N, Ben-Hail D, Porat Z (2013). Direct modulation of the outer mitochondrial membrane channel, voltage-dependent anion channel 1 (VDAC1) by cannabidiol: a novel mechanism for cannabinoid-induced cell death. *Cell Death Dis.*.

[129] Kaplan BL, Springs AE, Kaminski NE (2008). The profile of immune modulation by cannabidiol (CBD) involves deregulation of nuclear factor of activated T cells (NFAT). *Biochem. Pharmacol.*.

[130] Ignatowska-Jankowska B, Jankowski M, Glac W, Swiergel AH (2009). Cannabidiol-induced lymphopenia does not involve NKT and NK cells. *J. Physiol. Pharmacol.*.

[131] Rajesh M, Mukhopadhyay P, Batkai S (2007). Cannabidiol attenuates high glucose-induced endothelial cell inflammatory response and barrier disruption. *Am. J. Physiol. Heart Circ. Physiol.*.

[132] Casarejos MJ, Perucho J, Gomez A (2013). Natural cannabinoids improve dopamine neurotransmission and tau and amyloid pathology in a mouse model of tauopathy. *J. Alzheimers Dis.*.

